# Perioperative Hypothermia in Surgical Patients: A Retrospective Cohort Analysis at a Busy District General Hospital

**DOI:** 10.7759/cureus.70139

**Published:** 2024-09-25

**Authors:** Zain Habib, Mohammed Arifuzaman, Apurv Gupta, Neil Muscat, Sherif I Fawzy, Muhammad Umer Rasool, Ahmed Elbeltagi, Syed Ali Abbas Bilgrami, Muhammed Suneer Puthan Peedika, Sayan Bhattacharya

**Affiliations:** 1 Orthopaedics, North Manchester General Hospital, Manchester, GBR; 2 General Surgery, North Manchester General Hospital, Manchester, GBR; 3 Vascular Surgery, Manchester University NHS Foundation Trust, Manchester, GBR; 4 Surgery, North Manchester General Hospital, Manchester, GBR; 5 General and Colorectal Surgery, Manchester University NHS Foundation Trust, Manchester, GBR; 6 Internal Medicine, North Manchester General Hospital, Manchester, GBR

**Keywords:** anesthesia, core body temperature, hypothermia, perioperative hypothermia, surgery

## Abstract

Introduction

Perioperative hypothermia is a common yet underreported complication of surgery. It results from various factors, including cold operating theaters, anesthetic effects, environmental exposure, exposed tissues, and the administration of cold intravenous or irrigation fluids. This study aims to determine the incidence of perioperative hypothermia in a district National Health Service hospital to assess the feasibility of a randomized controlled trial (RCT) for interventions to prevent hypothermia.

Methods

This retrospective study included the data of 200 elective surgical patients at North Manchester General Hospital from June 1, 2022, to August 1, 2022. Inclusion criteria were elective general surgery, urology, breast, and gynecology patients aged 18 to 60 years. Exclusion criteria included emergency cases and patients younger than 18 or older than 60. Temperature measurement data were collected from the anesthesia records of the patients at six phases: preoperative, pre-induction, intraoperative, post-procedure, recovery room, and post-recovery. Data collection included specialty, surgery duration, and the use of intraoperative fluid warmers. Statistical analysis was performed using StatsDirect software (StatsDirect Ltd, Wirral, UK).

Results

Among the 200 patients, the overall incidence of hypothermia was 4% preoperatively, 5% pre-induction, 12% intraoperatively, 11% postoperatively, 8% in recovery, and 6% post-recovery. Intraoperative hypothermia incidence was significant, given that active warming was applied to patients with preoperative hypothermia. Regression analysis showed no correlation between intraoperative temperature and the use of intraoperative fluid warmers. Pre-induction temperature was the most statistically significant predictor of intraoperative hypothermia.

Conclusions

This study highlights the need for active interventions to recognize and prevent perioperative hypothermia in elective surgical patients. Active pre-warming of patients, regardless of surgery type and duration, is feasible and potentially beneficial. Future research should include an RCT comparing active and passive warming strategies to evaluate their effectiveness in improving perioperative outcomes.

## Introduction

Perioperative hypothermia, defined as a core body temperature of less than 36°C [1], is a recognized and underreported complication of surgery due to disrupted thermoregulatory mechanisms in anesthetized patients [2]. Perioperative hypothermia is reported in about 10% to 80% of patients [3-5]. This condition can result from various factors, including a cold operating room, anesthetic effects, environmental exposure, exposed tissues, and the administration of cold intravenous or irrigation fluids.

Thermoregulation

In healthy individuals, core body temperature is tightly maintained between 36.7°C and 37.1°C by the posterior hypothalamus, which receives inputs from heat and cold receptors located in the skin, deep tissues, spinal cord, brainstem, and hypothalamus [6]. Cold receptors are innervated by A-δ fibers, and warm receptors by C fibers, which communicate with the preoptic nucleus via the lateral spinothalamic tract or the trigeminal nerve for the head and neck. When body temperature deviates from the normal range, the hypothalamus initiates homeostatic and behavioral mechanisms to restore it. Responses to cold include vasoconstriction and shivering, while responses to heat include sweating and vasodilation [7].

Anesthesia and heat balance

Under general anesthesia, the behavioral response to temperature regulation, which requires conscious perception of body temperature, is severely impaired. Anesthetic agents reduce vasoconstriction and shivering thresholds, leading to vasodilation and heat loss from the core to the peripheries. Metabolic rate and heat production are reduced by 15% to 40% during general anesthesia [8]. Heat loss occurs through radiation (40%), convection (30%), evaporation (25%), and conduction (5%) [9]. Perioperative hypothermia can result in coagulopathy, sepsis, surgical site infection, prolonged drug effects, increased bleeding risk, myocardial ischemia, and adverse cardiac events [10].

Pre-warming involves warming the skin and peripheral tissues before anesthesia induction to reduce the central-to-peripheral temperature gradient, thus minimizing thermo-redistribution after anesthesia onset [5]. Passive warming, using warm cotton blankets, aims to maintain core body temperature by preventing heat loss to the ambient environment. Active warming, such as forced-air warming blankets, actively increases peripheral temperature, thereby raising core body temperature. Forced-air warming blankets circulate warm air in close contact with the patient's skin, increasing both peripheral and core body temperatures [11].

This study aims to determine the incidence of perioperative hypothermia at our busy district National Health Service (NHS) hospital. If significant, this incidence can serve as a feasibility study for a randomized controlled trial (RCT) to evaluate interventions to prevent perioperative hypothermia, particularly pre-warming. The study would compare pre-warmed elective surgical patients with those who are not pre-warmed, assessing perioperative hypothermia incidence, postoperative complications, and the length of hospital stay.

## Materials and methods

Our hospital follows the National Institute for Health and Care Excellence (NICE) guidelines, assessing all elective surgical patients for perioperative hypothermia and initiating interventions if necessary [1]. This retrospective study included 200 patients from North Manchester General Hospital from June 1, 2022, to August 1, 2022. Inclusion criteria were elective general surgery, urology, breast, and gynecology patients aged 18 to 60 years. Emergency cases and patients younger than 18 or older than 60 were excluded. This study was registered with the North Manchester General Hospital Clinical Audit and Quality Improvement (QI) team (registration number 10414). The data were collected retrospectively from the anesthesia records; hence, no ethics approval was required.

The data collected included preoperative, pre-induction, intraoperative, post-procedure, recovery room, and post-recovery temperatures. The preoperative temperature was the core body temperature measured one hour before anesthesia [1]. Pre-induction temperature was recorded immediately before anesthesia induction. Intraoperative temperature was measured 30 minutes after anesthesia induction. Post-procedure temperature was recorded at the end of surgery and anesthesia. Recovery room temperature was measured after patients were shifted from the operating room, and post-recovery temperature was recorded upon transfer to a ward from recovery. Temperatures were measured using tympanic or temporal thermometers.

All patients who developed perioperative hypothermia were managed according to the NICE guidelines CG65 [1]. This included active warming for at least 30 minutes before the induction of anesthesia, maintaining active warming throughout the intraoperative phase, and using intravenous fluids (500 mL or more) and blood products warmed to 37°C with a fluid warming device. Hypothermia in these patients was readily reversed, and no surgeries were terminated due to severe hypothermia.

Data on the specialty, surgery duration, and use of intraoperative fluid warmers were collected. Data collection was done manually with the help of the audit team. Statistical calculations were performed using StatsDirect software (StatsDirect Ltd, Wirral, UK) from Manchester University NHS Foundation Trust. Multivariate regression analysis was conducted, with p < 0.05 considered significant. Regression analysis was performed to determine predictor values for our results, guiding future RCT planning.

## Results

**Figure 1:**
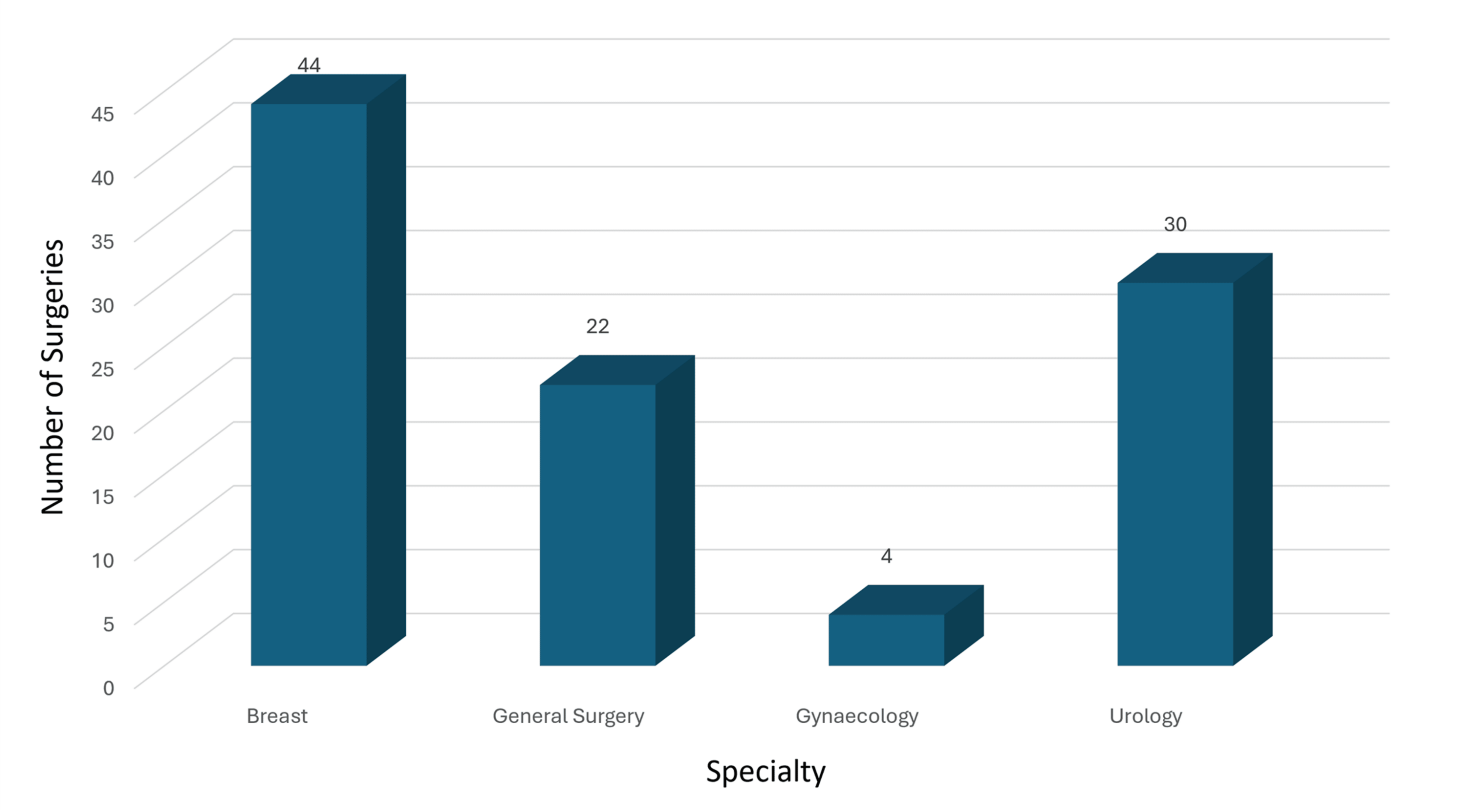
Incidence of hypothermia per specialty

**Figure 2:**
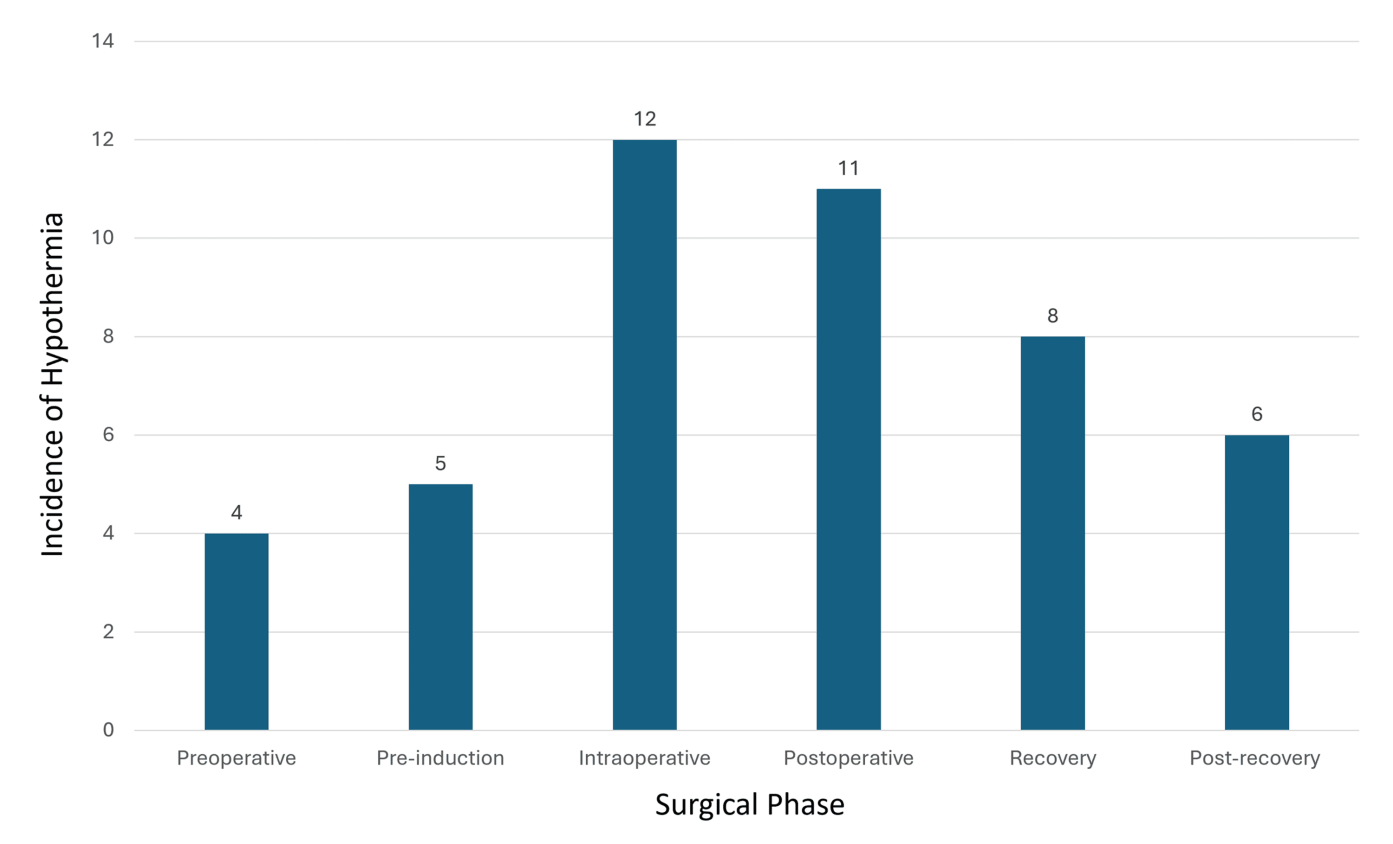
Incidence of hypothermia in different phases of surgery

**Figure 3:**
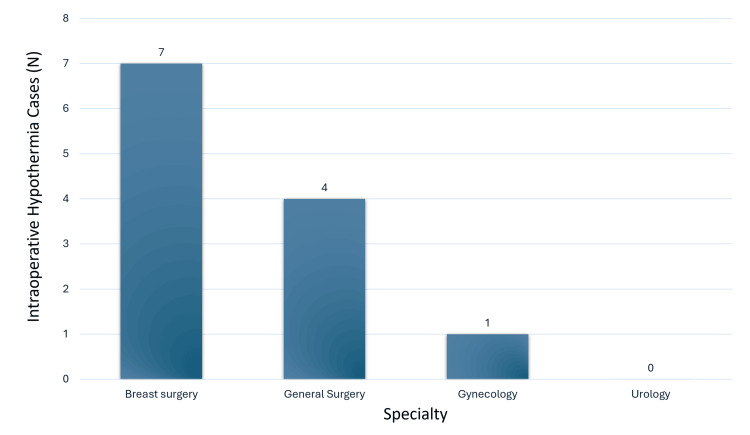
Intraoperative hypothermia incidence per specialty

Of the 200 patients, 88 underwent breast surgery, 44 underwent general surgery, eight underwent gynecology procedures, and 60 underwent urology procedures (Figure 1). We calculated the overall incidence of hypothermia for each phase of surgery. Preoperative hypothermia occurred in 4% of patients, pre-induction hypothermia in 5%, intraoperative hypothermia in 12%, postoperative hypothermia in 11%, recovery hypothermia in 8%, and post-recovery hypothermia in 6% (Figure 2). The intraoperative hypothermia incidence of 12% was significant, considering that patients with preoperative hypothermia were actively warmed before anesthesia induction. The incidence per specialty is presented in Figure 3: breast surgery had 14 cases (58.3%), general surgery had eight cases (33.3%), gynecology had two cases (8.3%), and urology had zero cases.

**Figure 4:**
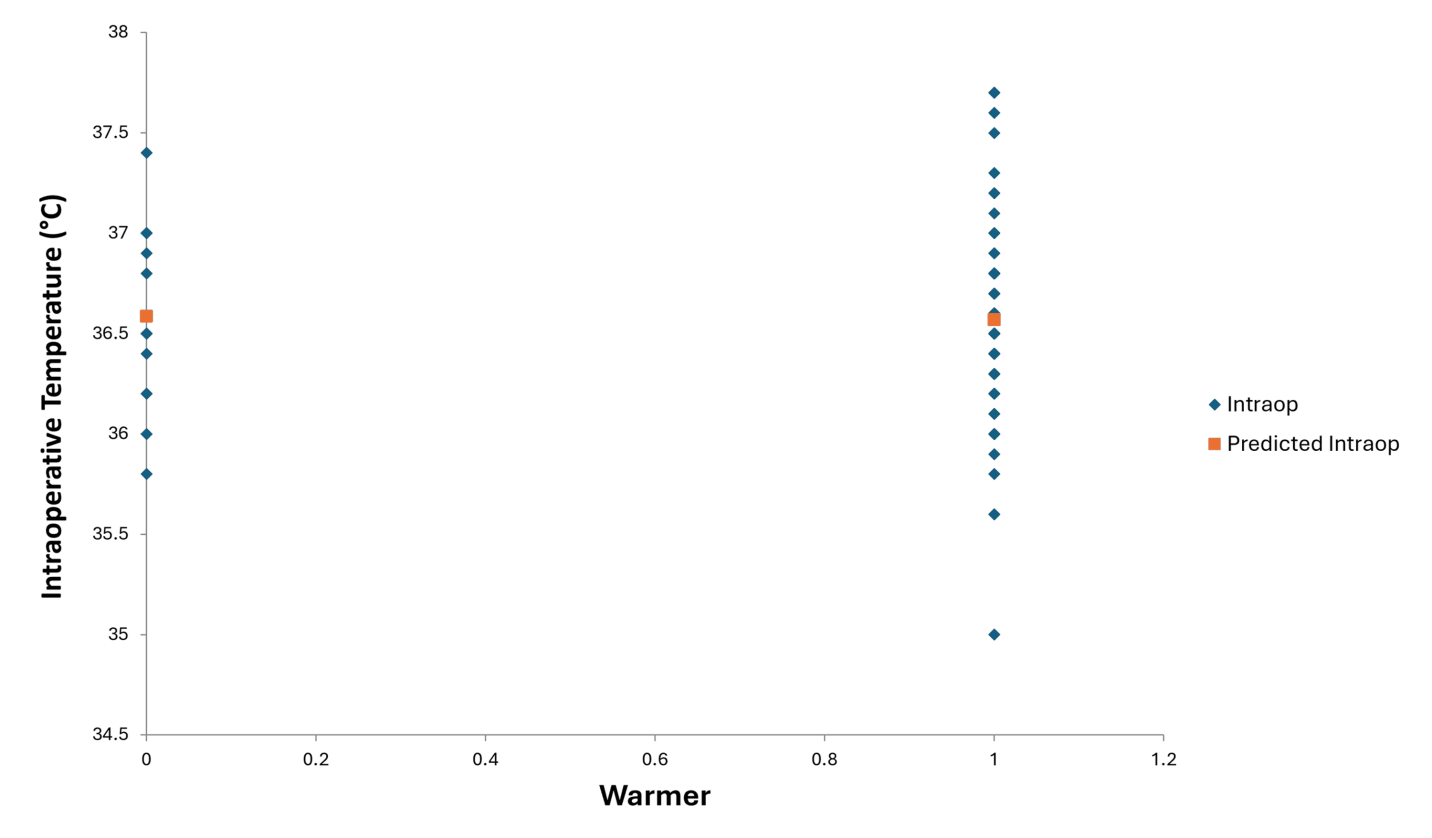
Line fit plot for the intraoperative patient core body temperature and use of intraoperative fluid warmers

**Figure 5:**
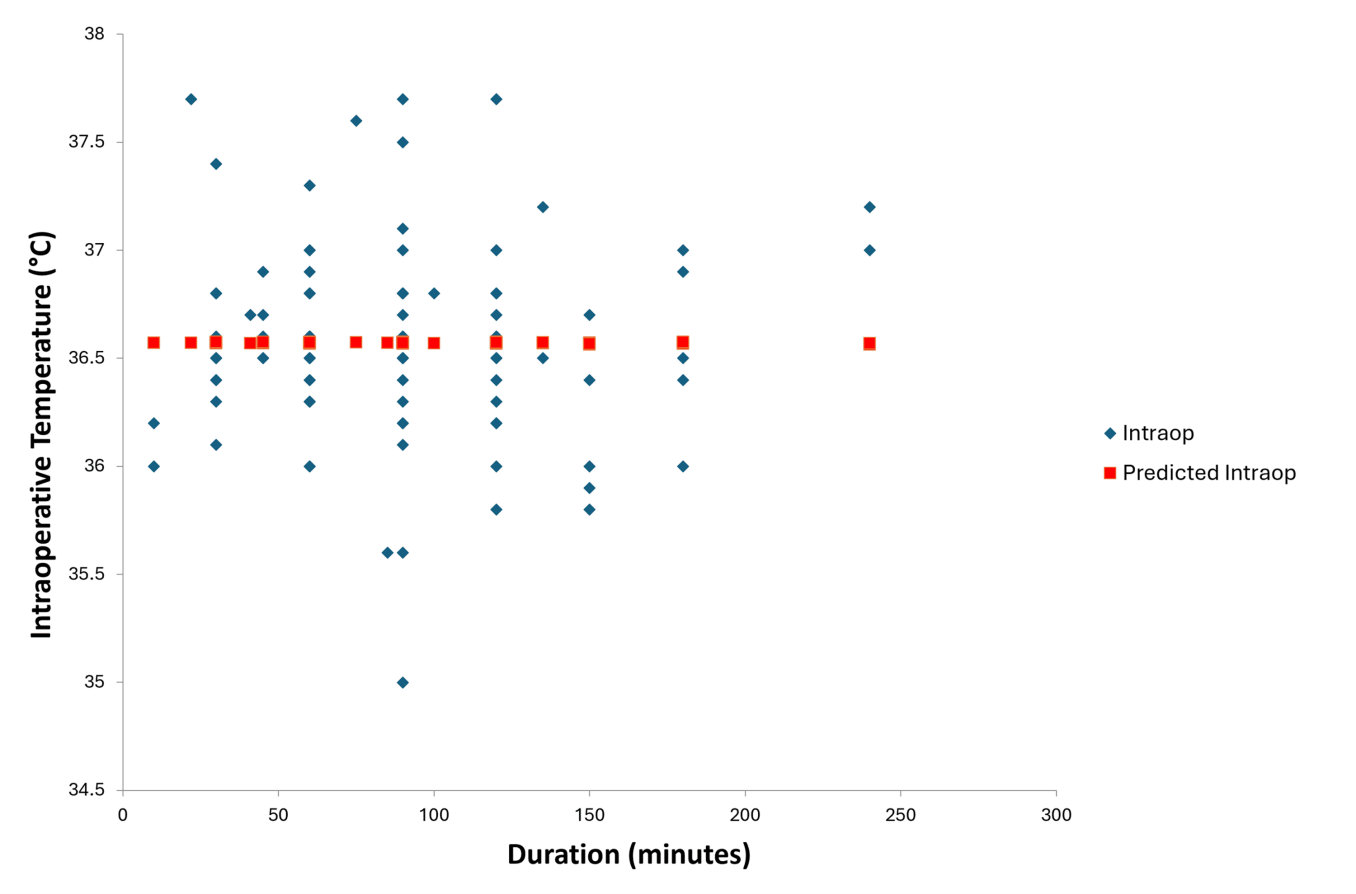
Line fit plot after regression analysis between duration of surgery in minutes and intraoperative patient temperature

Regression analysis of intraoperative temperature measurements and the use of intraoperative fluid warmers resulted in a p-value of 0.90, indicating no correlation between the two in our study. The plot of Figure 4 shows that patients developed intraoperative hypothermia even while using intraoperative fluid warmers. The duration of the study did not show any statistical significance. Regression analysis of intraoperative temperature measurements and the duration of surgery resulted in a p-value of 0.95 (Figure 5).

**Figure 6:**
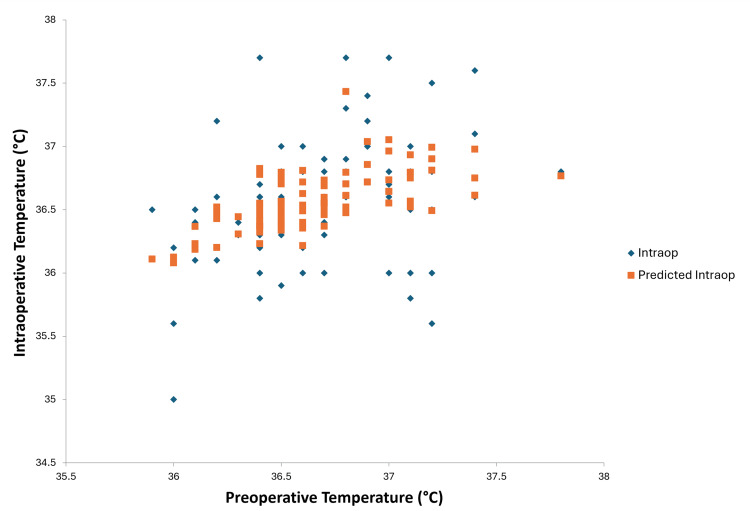
Line fit plot after multiple regression analysis between preoperative and intraoperaive temperatures

**Figure 7:**
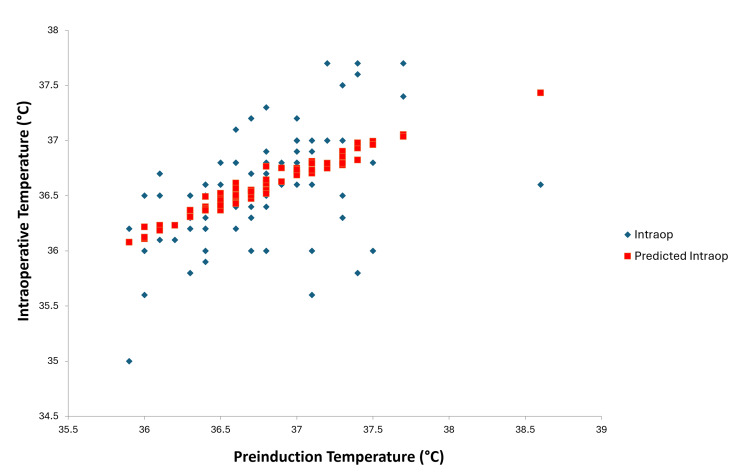
Line fit plot after multiple regression analysis between pre-induction and intraoperative temperatures

Multiple regression analysis of preoperative temperatures, pre-induction temperatures, and intraoperative temperatures resulted in a p-value of 0.237 for preoperative temperature (Figure 6) and <0.001 for pre-induction temperature (Figure 7). This suggests that pre-induction temperature was the most statistically significant parameter for predicting intraoperative hypothermia. The regression analysis also predicted intraoperative temperature based on pre-induction temperature. The mean pre-induction temperature for all 100 patients was 36.75°C. The regression analysis line fit plot between pre-induction temperature and intraoperative temperature predicts that if the mean pre-induction temperature is raised to 37.2°C, the predicted intraoperative temperature will be 36.74°C.

## Discussion

Perioperative hypothermia prevention lacks clear protocols, as there is evidence that suggests warming irrigation fluid has no statistical significance in the prevention of intraoperative hypothermia between room temperature and warmed fluid groups [12], although clinical practice guidelines recommend that pre-warmed irrigation fluid of 38-40°C is effective [13]. A further systematic review suggests that warm intravenous and irrigation fluids are effective in the prevention of perioperative hypothermia compared to the non-warmed group [14]. Our study found no statistically significant correlation between the use of warmed intravenous fluids and the incidence of perioperative hypothermia, which may be associated with a smaller cohort of patients and the fact that all patients received warmed intravenous fluids.

There are many risk factors associated with perioperative hypothermia, including ambient theatre temperature of the operating room, shock, spinal cord injury, adrenal disease, cardiac dysfunction, cold and wet clothing, comorbidities, cold intravenous fluids and blood products, duration of general anesthesia greater than three hours, epidural and spinal anesthesia, and low preoperative temperatures [13,15-18].

Early identification of patients at risk of perioperative hypothermia, and prevention must be initiated one to two hours prior to surgery, and intraoperative temperature measurement must be taken at least every 15 minutes [16,19].

Prevention and management of perioperative hypothermia primarily depends on two methods.

Passive patient warming

This is a means to reduce radiation and convective heat loss through the skin, which is effective in mild hypothermia [16,20]. This can be achieved by the use of warm blankets, removal of wet clothes, shoes, and socks, and the use of a heat and humidity exchanger.

Active patient warming

This is a method where direct heat is transferred to the patient, which helps to raise as well as maintain the core body temperature [13]. This can be achieved by the use of infrared light, electric blankets, mattresses or blankets with warm water circulation, or convective air warming transfer, and the use of warm irrigation and intravenous fluids [12,21,22].

Both strategies have been shown to be effective in mild hypothermia [23], and rewarming is recommended in patients with severe hypothermia [13,16,23].

Campbell et al. reported that warm intravenous fluids keep patients warmer during surgery than at room temperature [6], but we did not find any statistical correlation between warm intravenous fluids and the incidence of perioperative hypothermia in our patients. All the patients had received warmed intravenous fluids, and 12% developed intraoperative hypothermia.

Intraoperative warming is effective in preventing intraoperative hypothermia [6,13,24]. In our study, all the patients were elective surgical patients who underwent general anesthesia with intraoperative warming, and 12% developed hypothermia. Active pre-warming of patients 20-30 minutes before surgery has been shown to reduce the incidence of perioperative hypothermia [19,24]. In our study, only the patients who were hypothermic in the pre-anesthetic phase were actively pre-warmed, which was 5% of the patient population.

Limitations

This study is limited by the small number of patients included, and a power calculation was not performed to determine the sample size. As a feasibility study, it aims to understand the dynamics of perioperative hypothermia and assess whether intervention is warranted. The inclusion of varied cases from different specialties weakens the study's ability to make specific recommendations for any particular specialty regarding the prevention of perioperative hypothermia. Urology procedures typically required less patient and tissue exposure than breast, general surgery, and gynecology procedures, potentially skewing the data. Breast surgery often necessitated exposing both the breasts and axillae, leading to significant heat loss, even though abdominal and lower body warmers were used.

Postoperative complications due to hypothermia, the impact on the length of hospital stay, and the duration of postoperative recovery were not assessed. The temperature measurements were obtained using both tympanic and temporal thermometers, which could introduce variation in the recordings. The study also did not account for the volume of warmed fluid used or the intraoperative steps for hypothermia correction. The room temperature of the preoperative ward was not recorded, which may have influenced patients' pre-induction temperatures.

Surgeries were performed in different operating theaters simultaneously, as there are separate theaters for general surgery, gynecology, urology, and maxillofacial surgery running in parallel on any given day. This could lead to data discrepancies, as the ambient temperature of each theater varies, affecting intraoperative heat loss and patient temperature. The intraoperative use of different warming devices was not calculated, which is critically significant and warrants further investigation in future studies.

Future studies

This study has the potential to constitute an RCT to compare the effects of preoperative warming in elective patients. The goal will be to compare active and passive warming and investigate the feasibility of active warming (forced-air warmer) as a perioperative warming strategy. Future research will involve a prospective, open-label, single-blind RCT pending local ethics committee approval. We aim to study a cohort of electively listed surgical patients, randomizing them into intervention and control groups. The intervention group will be actively warmed to reach a pre-induction temperature of 37.2°C using a forced-air warming device throughout the perioperative period. The control group will be managed according to local hospital protocols without active warming unless necessitated by preoperative hypothermia.

## Conclusions

This study highlights the need for active interventions to recognize and prevent perioperative hypothermia. The incidence of hypothermia in elective surgical patients indicates that more can be done to prevent this condition. The study suggests that active pre-warming of all elective patients, regardless of the type and duration of surgery, is feasible and potentially beneficial. Future research should include an RCT comparing active and passive warming strategies. The goal is to determine if active warming, using a forced-air warming device, is more effective than passive warming. Such a trial will assess outcomes, including core body temperature, patient thermal comfort, length of stay in the post-anesthesia care unit, and overall hospital stay.
